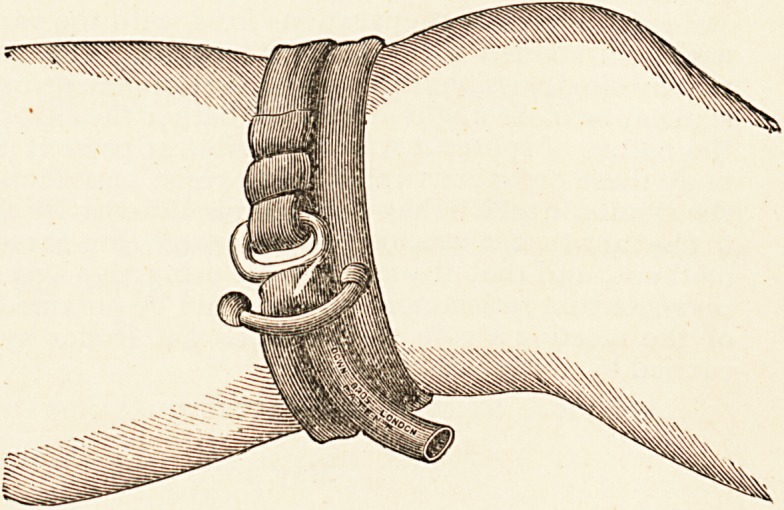# Notes on Preparations for the Sick

**Published:** 1893-06

**Authors:** 


### Notes on Preparations for the Sick.

**Bynin Liquid Malt. Byno-Pancreatin. Byno-Pepsin. Byno-Hypophosphites. Coca-Bynin.**—ALLEN & HANBURYS, London.—These preparations have as their basis a concentrated *liquid* extract of malt. They are therefore more pleasant to take, inasmuch as they are free from the troublesome viscidity of the ordinary malt extracts.

**Thyroid Extract.**—BRADY & MARTIN, Newcastle-on-Tyne.—The utility of the thyroid gland was abundantly shown in our Report on Medicine in the number for March. It is clear that cases of myxœdema must be given the benefit of treatment by thyroid gland, or the preparations therefrom. Messrs. Brady and Martin supply the fluid extract in two forms: one for administration by the mouth, and the other for use hypodermically. The extract is also prepared as a powder, which has the advantage of being more stable.

---

**Tabloids of Ichthyol; Resorcin; Ergotin; and Blaud's Pill. Peptonic Tabloids. Sugar-coated Tabloids.**—BURROUGHS, WELLCOME & Co., London.—**Ichthyol** Tabloids contain 2½ grains of the sodium sulpho-ichthyolate, and are coated with sugar. They are readily soluble, and the disagreeable flavour and odour of the drug are completely concealed. It is said that ichthyol by extracting oxygen from the tissues becomes a reducing agent, producing an invigorating and antiseptic influence throughout the body and an astringent effect on the blood vessels, and hence that it is of immense value in the internal treatment of those diseases in which hyperæmia and enlargement of capillaries occur, more especially in acne rosacea, chronic rheumatism, chronic catarrh of the stomach, and other chronic affections of the skin and mucous surfaces. The drug, derived from the distillation of a bituminous quartz found in the Tyrol, and deriving its name from its origin in what is believed to be the remains of decomposed animals and fish, has no agreeable properties to recommend it, and would be inadmissible as an internal medicine but for its elegant preparation in the form of a sugar-coated tabloid. In this form the many virtues which it is said to have may be amply verified.

**Resorcin** Tabloids contain three grains of the drug: they are soluble, and convenient for the preparation of solutions for topical application in cases of lupus, epithelioma, or cancer. They are said to be of use in cases of fermentative dyspepsia and consequent diarrhœa. As an intestinal antiseptic they should be of service, and if given frequently would also prove to be an efficient antipyretic agent.

**Ergotin** Tabloids contain three grains of the purified extract of ergot, and they are sugar-coated. They are prepared with care, and may be relied upon to have the full therapeutic activity of the best preparations of this popular drug.

**Blaud's Pill** Tabloids contain four grains of the carbonate of iron in an unoxidised condition. Upon wetting the surface the green colour of the ferrous carbonate is at once manifest. The ingredients of the tabloids do not decompose till after the dose has been swallowed, when the ferrous carbonate in an active state is produced.

**Peptonic** Tabloids contain internally zymine with lactophosphate of lime: this combination is coated over with keratin. Pure pepsine is then added, and the completed tabloid is covered over with white sugar. The pepsine peels off in the stomach, and the other ingredients pass into the intestine intact to assist in the work of pancreatic digestion. Such a tabloid as this must give the zymine a better chance of displaying functional activity than when, being taken in the ordinary way, it has to run the risk of becoming inert in the stomach.

**Sugar-coated Tabloids**, containing various drugs, are now supplied: they are promptly soluble, easy to swallow, and efficient in their action.

---

**Malto-Carrageen. Maltine with Lime.**—MALTINE MANUFACTURING CO., London.—The first of these should be useful in chronic pulmonary affections: it combines the nutritive and demulcent properties of maltine and carrageen, with the active principle of an efficient expectorant—yerba santa. The second contains hypophosphites of lime, soda, and iron, in combination with the concentrated extract of various cereals commonly known as maltine.

---

**Peptonised Beef Wine, with Coca. Peptonising Tablets.**—T. BUXTON, Clifton.—The beef peptone, with coca wine, must be a valuable restorative, and is a useful kind of medicine in convalescence from acute diseases and in conditions where more active medication is not necessary.

The peptonising tablets are very convenient for preparing half-pints of peptonised milk for immediate consumption. For larger quantities more tablets than one will be needed, and the product should be boiled when not required for immediate use. We have found them to be convenient and effective.

---

**Zinc-Gelatin.**—J. F. EARDLEY, Sheffield.—This, suggested by Mr. Dale James, is a preparation of zinc and gelatine, of a firm consistence. The tin in which it is sent out is placed in a saucepan of hot water, and the softened preparation can then be applied to a patient's back with a brush, or dabbed on with cotton wool. It forms a firm and impenetrable covering for the skin, and prevents bed-sores in patients who are constantly wet.

---

**Samways' Tourniquet Clip** (Anchor Pattern).—DOWN BROS., London.—This most useful contrivance consists of a piece of rubber tubing, furnished with a steel clip shaped like an anchor at one end. It can be applied in the easiest possible manner by first passing the stretched rubber once or twice round the limb, and then beneath one of the anchor flukes, over the shank, and back beneath the other fluke. Soft rubber tubing is now universally acknowledged to make the best tourniquet, and the anchor clip seems to make a simple and effectual fastening.

---

**Gem Menthol Inhaler.**—T. Christy & Co., London.—This is a most compact but efficient instrument. It can be readily carried in the waistcoat-pocket, and is always ready for use. At a temperature of 50° F., or less, menthol does not volatilise rapidly enough to give good results; but if the inhaler is held in the hand for a few moments the menthol volatilises freely. The inhaler becomes strongest when carried in the pocket, which keeps it at a temperature of nearly 98° F.

## Figures and Tables

**Figure f1:**
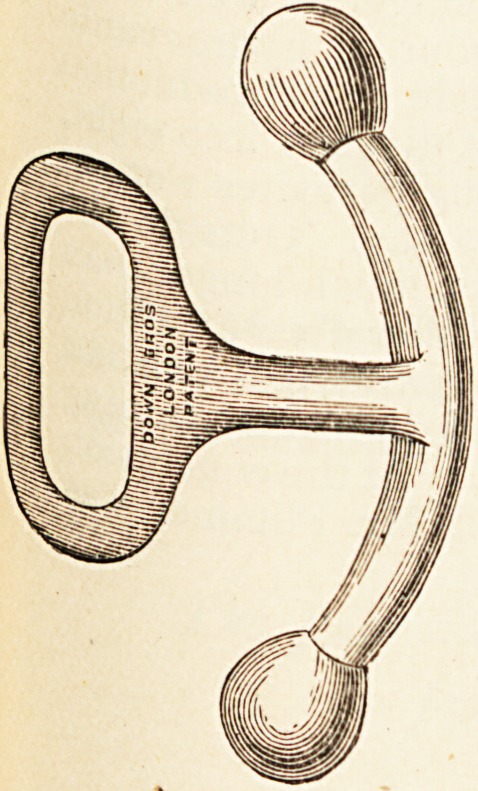


**Figure f2:**